# 

**Published:** 1844-06

**Authors:** 


					Yolnme IV.
J CM B, 1844,
No. 4.
THE
mmffi
?*10.
j
OF
Btitntt.
mmm
PUBLISHED UNDER THE AUSPICE^ OF THE
D. P. S.
, M.D., D.D. S.
/ M. D., D. fi. S.
B ALTIMOB E :- A IIMSTKONG ItB E R R Y,
iiTHTiWiiva
WOODS AND CRANE, PRS., HALT.
$5 OO per annum, payable in advance.

				

